# A systematic review on *in vitro* 3D bone metastases models: A new horizon to recapitulate the native clinical scenario?

**DOI:** 10.18632/oncotarget.8394

**Published:** 2016-03-26

**Authors:** Francesca Salamanna, Deyanira Contartese, Melania Maglio, Milena Fini

**Affiliations:** ^1^ Laboratory of Biocompatibility, Technological Innovation and Advanced Therapy, Rizzoli RIT, Rizzoli Orthopedic Institute, Bologna, Italy; ^2^ Laboratory of Preclinical and Surgical Studies, Rizzoli Orthopedic Institute, Bologna, Italy

**Keywords:** systematic literature review, bone metastases, 3D in vitro model, cancer cells, metastatic microenvironment

## Abstract

While the skeleton is not the only organ where metastasis can occur, it is one of the preferred sites, with a significant impact in patients' quality of life. With the aim of delineating the cellular and molecular mechanisms of bone metastasis, numerous studies have been employed to identify any contributing factors that trigger cancer progression. One of the major limitations of studying cancer-bone metastasis is the multifaceted nature of the native bone environment and the lack of reliable, simple, and not expensive models that strictly mimic the biological processes occurring *in vivo* allowing a correct translation of results. Currently, with the growing acceptance of *in vitro* models as effective tools for studying cancer biology, three-dimensional (3D) models have emerged as a compromise between two-dimensional cultures of isolated cancer cells and the complexity of human cancer xenografts in immunocompromised animal hosts. This descriptive systematic literature review summarizes the current status of advanced and alternative 3D *in vitro* bone metastases models. We have also reviewed the strategies employed by researchers to set-up these models with special reference to recent promising developments trying to better replicate the complexity and heterogeneity of a human metastasis *in situ*, with an outlook at their use in medicine. All these aspects will greatly contribute to the existing knowledge on bone metastases, providing a specific link to clinical scenarios and thus making 3D *in vitro* bone metastasis models an attractive tool for multidisciplinary experts.

## INTRODUCTION

Metastasis, defined as the spread and growth of tumor cells to distant organs, is a dejected consequence of many types of tumors, representing the most devastating attribute of cancer [1–4]. Bone tissue is the third most frequent site for metastatic disease (after lung and liver) [5–10], causing severe pain, pathologic fractures, life-threatening hypercalcemia, spinal cord compression, immobility and, ultimately, death in patients afflicted with advanced breast, prostate, lung, kidney and thyroid cancers [11–16]. The process of cancer metastases, following tumor growth at the primary site of origin, involves intravasation and survival in the bloodstream, arrest, extravasation and finally establishment, by invasion and angiogenesis, at a distant site [17–18]. Tumor cells that metastasize in bone induce destructive osteolytic and/or bone forming osteoblastic lesions [12, 19] and ‘teach’ this affected bone microenvironment to produce factors that stimulate tumor cell growth [20–21]. In general, once bone metastases are present, patient survival is dramatically reduced. Most patients with metastatic bone disease survive for 6-48 months following diagnosis. Thus, significant effort has focused on understanding the mechanisms driving tumor dissemination to bone. However, the understanding of the cellular and molecular pathways involved in cancer-bone interaction and in metastasis treatment needs reliable models able to mimic the biological processes occurring in patients.

The structure, function and remodeling of bone as an organ is dynamically retained by three-dimensional (3D) interactions among several cell types, including those of osteoblastic, osteoclastic, endothelial and hematopoietic lineages [22–25]. In order to investigate the mechanisms underlying metastatic processes in bone, a variety of two-dimensional (2D) cell cultures and *in vivo* animal models have been developed and used [26–31]. It is well known that cell-cell and cell-matrix interactions have a key role in tumor morphogenesis and cancer metastasis. However, there is no ideal model able to mimic *in vitro* all these *in vivo* physiological events. Conventional 2D cell culture models have brought great insight into the ability of tumor cells to grow, but they do not provide any information about the complex interactions between cancer cells and the physicochemical environment that exists within living tumors [32–33]. In addition, superimposed spatial cues, including substrate depth and cell connectivity, limit the applicability of 2D culture for testing pharmacologically active compounds. Such limitations may provide less reliable data leading to restrictions for the translation of results into clinical applications. *In vivo* animal models have more relevance and overcome many of the limitations of 2D models but, in addition to the high costs and systemic complexity, they often do not faithfully reproduce the biological programs specific of the human species and fail to be predictive of a clinical outcome [34]. To date, there are several established *in vivo* animal models of skeletal metastasis, varying in host animal, type of cancer investigated, method of tumor inoculation and metastatic potential. In general, three broad approaches have been employed to investigate bone metastasis *in vivo*. These include syngeneic (spontaneous, inducible or transplantable) models, genetically engineered models and xenograft models [35–37]. Each of these models has inherent advantages and limitations that can have a significant impact on the clinical relevance of the generated data. In addition, *in vivo* models have a high ethical impact and it is therefore necessary to bridge the gap between 2D cultures and animal models. More recent studies have attempted to overcome the limitations of both systems by the development of 3D *in vitro* models that can be tailored to be biomimetic and accurately reproduce the native physiological scenario of metastases. The aims of biomimetics 3D models include, but are not restricted to, 1) providing proper matrix components in a 3D configuration as found *in vivo*, 2) culturing cancer cells, endothelial cells and other associated cells in a spatially relevant way, 3) monitoring and controlling hypoxia in order to mimic the levels found in physiological tumors conditions and 4) monitoring the release of angiogenic factors by cancer cells in response to hypoxia [38]. Thus, all these aspects mean that 3D *in vitro* models can be personalized in order to mimic different stages of cancer progression: from the initial development to metastasis. However, despite the growing awareness of the importance of 3D culture system in cancer research, few attempts have been undertaken to create such models in order to answer biological questions about bone metastasis.

This descriptive systematic literature review aims at reviewing current 3D *in vitro* models of bone metastases and the strategies employed by researchers to set-up these models. In particular we will look at recent promising developments that try to mimic more closely the metastatic microenvironments, providing a compromise between the limited approach of 2D monolayer isolates cancer cells, and the complexity of growing human tumors in xenogeneic hosts. Although every “model” is imperfect by definition, it can still be useful if it offers controlled conditions in which a given hypothesis can be evaluated. It is crucial that researchers are familiar with the advantages and disadvantages of specific models, as only then they can work around these parameters [39].

## MOTIVATIONS

### Why a systematic review?

We believed there was the need of a descriptive systematic literature review of 3D bone metastasis *in vitro* models in order to understand which would be the most successful and promising model that can recapitulate the clinical scenario and would provide researchers, designers, and practitioners with a starting point for advancing in this field. Our aim is to provide answers to questions such as: “Since bone is a complex environment containing many cell types, is it possible to study all the mechanisms of bone metastases in a 3D *in vitro* model?”, “What happens when bone metastatic cancer cells are added to a 3D *in vitro* model?”, “What kind of 3D *in vitro* model should be used and what model closely mimics the clinical scenario?”, “Are these models able to catalyze the development of new therapeutic interventions?”, “How much the proposed model closely reflects the data collected so far in clinical trials?” and “How much the proposed model can help in elucidating the mechanisms at the basis of bone invasion and metastasis?”.

More in detail, we want to organize the knowledge accumulated in nearly 10 years of research, learn from previous studies that used different 3D *in vitro* models of bone metastasis, and build a foundation for future models, as we believe there is an urgent need for clinically relevant experimental models suitable for the study of bone metastases.

## MATERIALS AND METHODS

### Descriptive systematic literature review

Our descriptive literature review involved a systematic search that was carried out, according to the Preferred Reporting Items for Systematic Reviews and Meta-Analyses (PRISMA) statement, in three databases (www.pubmed.org, www.scopus.com, www.webofknowledge.com). The keywords were: (bone metastasis OR cancer bone metastasis) AND (three dimensional OR 3D OR 3d) AND (culture OR co-culture OR model OR system OR *in vitro* culture OR *in vitro* co-culture OR *in vitro* model OR *in vitro* system). We sought to identify studies where 3D *in vitro* models of bone metastases were employed. Publications from 2005 to 2015 (original articles in English) were included. Additional studies that were not found by our initial search were identified analyzing the reference lists from the included articles. A public reference manager (“www.mendeley.com”) was used to delete duplicate articles.

## RESULTS

**Figure 1:**
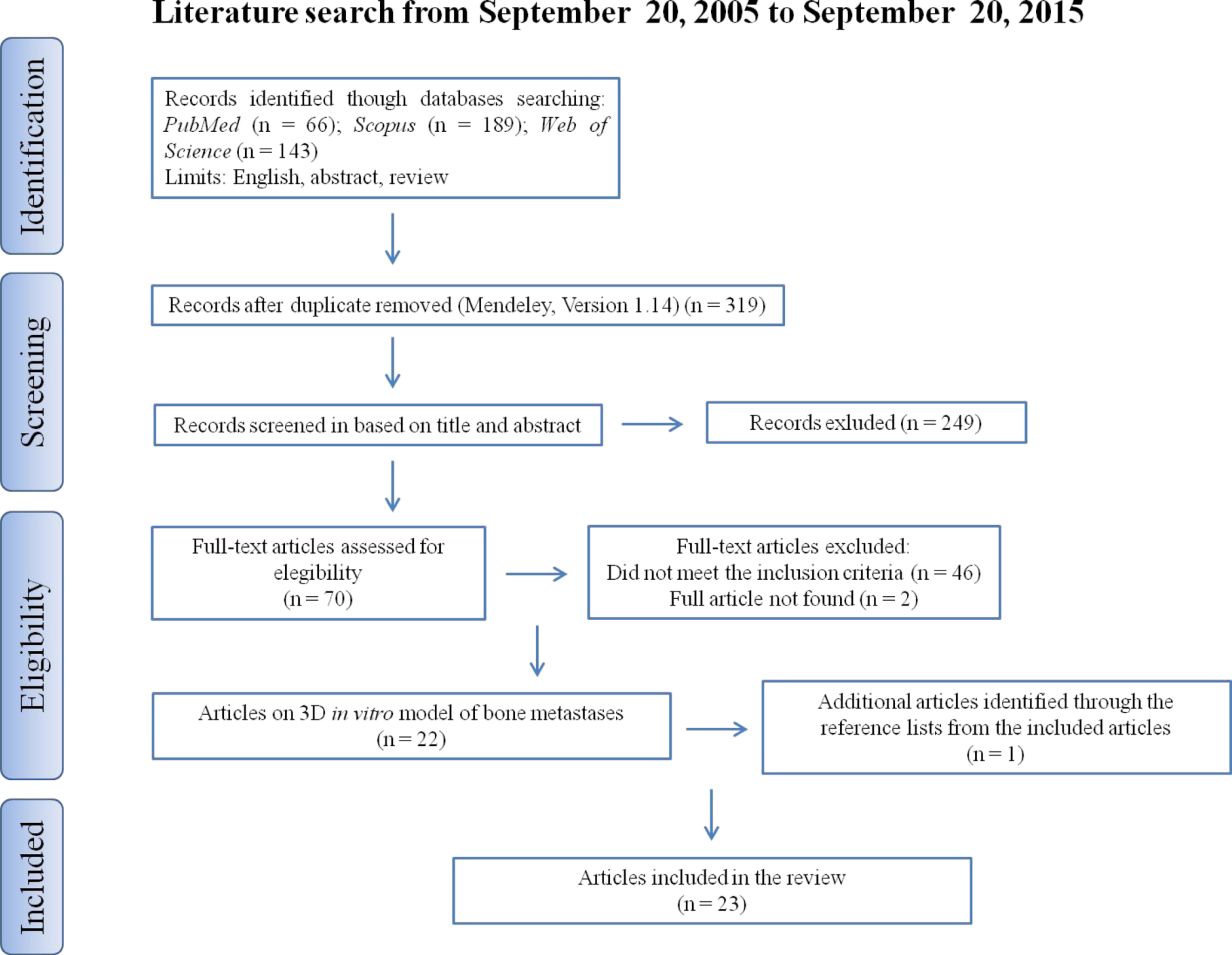
Systematic literature review flow diagram

**Figure 2:**
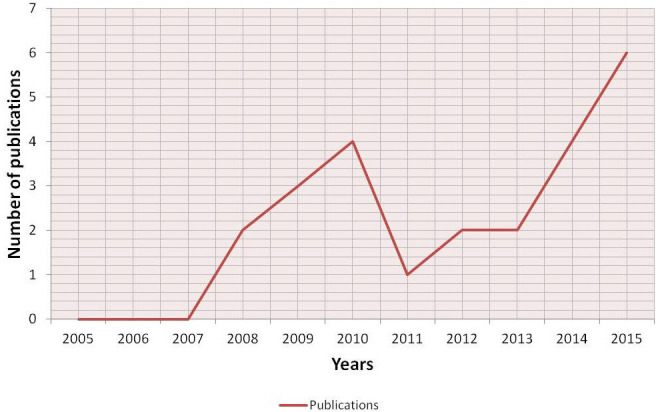
Historical distribution of 3D bone metastases *in vitro* model works according to the year of publication

An initial literature search retrieved 398 references (Figure 1); 66 articles were identified using www.pubmed.org, 189 articles using www.scopus.com and 143 articles were found in www.webofknowledge.com. Subsequently, the resulting references were submitted to a public reference manager (Mendeley 1.14, “www.mendeley.com”) to eliminate duplicate articles. Of the 319 remaining articles, 70 publications were selected for supplementary analysis based on the title. Abstracts and complete articles were then reviewed to establish whether the publication met the inclusion criteria. After this screening process 22 articles were recognized eligible for the review (Figure 1) considering publications from 2005 to 2015 (Figure 2). From the reference lists of the selected articles, 1 additional publication, not found by our initial search, was recognized (Figure 1). We did not perform meta-analyses of the selected studies, but reported the results in a descriptive fashion.

**Figure 3:**
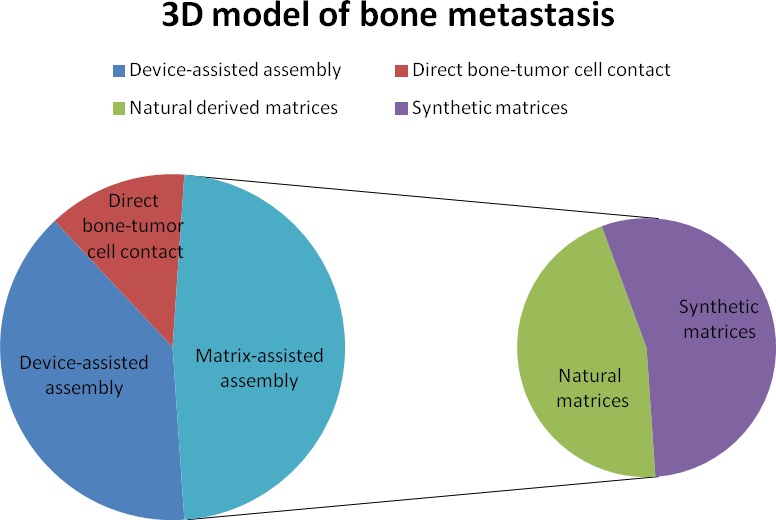
**A.** Pie chart of bone metastases studies that considered different 3D models: device-assisted assembly, matrix-assisted assembly and direct bone-tumor cell contact. **B.** Pie chart of the number of studies using natural or synthetic matrices.

**Figure 4:**
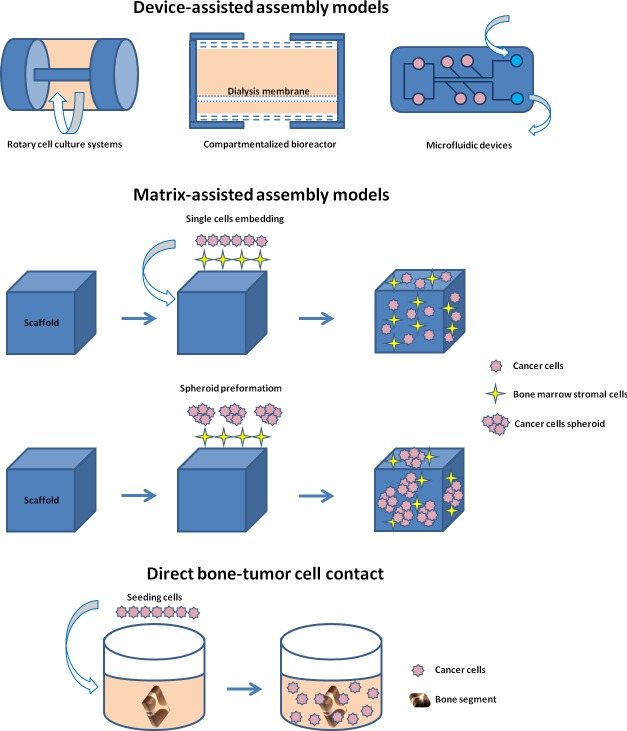
Schematic representation of the different 3D models of bone metastasis emerging from this review: device-assisted assembly models, matrix-assisted assembly models and direct bone-tumor cell contact models

By considering the different 3D models of bone metastasis emerging from this review, we stratified the papers according to: device-assisted assembly models, matrix-assisted assembly models and direct bone-tumor cell contact models (Figures 3 and 4).

**Figure 5:**
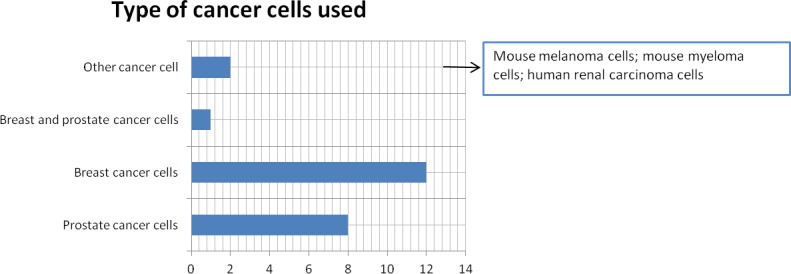
Cancer cell type used to set-up 3D bone metastasis models

Although the topic of the study was not to evaluate the type of cancer cell used to set-up the different models, we found that the majority of the reviewed papers used prostate cancer cells or breast cancer cells (Figure 5).

## DEVICE-ASSISTED ASSEMBLY OF BONE METASTASIS MODELS

In order to increase the relevance of an *in vitro* bone metastasis model, the recreation of its cellular architecture and its microenvironment is fundamental. Moreover, any physicochemical environmental parameter known to influence drug response, such as temperature, pH and oxygen, should be recreated using tissue engineering bioreactors and microfluidic systems (or micro-bioreactors) (Table 1).

**Table 1:** 3D device-assisted assembly models of bone metastasis

3D model	Cancer cell type	Experimental design	Main results	Advantages	Disadvantages	Reference
Rotating wall vessel (RWV) bioreactor	Prostate cancer cells	Co-culture of human osteosarcoma cell line, normal immortalized human osteoblastic cell line with LN and C4-2 prostate cancer cells and derivate bone stromal cells.	Normal and osteosarcoma bone stromal fibroblast co-cultured with C4-2 underwent permanent morphological changes.	- Forges direct interaction between prostate cancer cells and bone stromal cells; - Easily reproducible.	- Absent 3D matrix interaction; - No similarities with native bone microenvironments; - No hypoxic setting.	Sung et al.2008
Bioreactor system	Breast cancer cells	Co-culture of murine derived OT with MDA-MB-231 metastatic breast cancer cells.	MDA-MB-231 breast cancer cells form colonies which were able to penetrate the murine OT.	- Murine OT generated from bioreactor showed bone tissue like characteristics; - Able to detect cell migration and matrix remodeling.	- Difficult to separate the different cell types; - 3D cultures grow for several months; - No hypoxic setting; - Difficult to reproduce.	Dhurjati et al 2008; Mastro et al. 2009; Krishnan et al. 2011
Bioreactor system	Breast cancer cells	Co-culture of mineralized collagenous osteoblastic tissue with osteoclasts followed by reinfusion with proliferating pre-osteoblasts and MDA-MB-231 breast cancer cells.	Osteoclasts, differentiated in the presence of osteoblasts, led to degradation of the collagen-rich extracellular matrix. Addition of metastatic breast cancer cells to the co-culture mimicked the vicious cycle.	- Detection of cell migration and matrix remodeling.	- Difficult to separate the different cell types; - 3D cultures grow for several months; - No hypoxic setting; - Difficult to reproduce.	Krishnan et al. 2014
Bioreactor system	Melanoma cells, human prostate cells and mouse myeloma	Co-cultures of mouse melanoma cells, human prostate cells and mouse myeloma cells with MC3T3-E1 osteoblasts.	Each of these different lines displayed characteristic growth patterns.	- Detection of cell migration and matrix remodeling.	- Difficult to separate to different cell types; - 3D cultures grow for several months; - No hypoxic setting; - Difficult to reproduce.	Krishnan et al. 2015
Microfluid system	Breast cancer cells	Tri-culture of osteodifferentiated hBM-MSCs, HUVECs and MDA-MB-231 breast cancer cells.	The model allows to quantify tumor cell extravasation and micrometastasis generation within a bone-like microenvironment.	- Biochemically and biophysically controlled 3D microenvironments.	- Vascular wall represented by an endothelial monolayer; - Difficult to separate different cell types; - No similarities with native bone microenvironments; - Absent 3D cell-matrix interaction; - No hypoxic setting; - Difficult to reproduce.	Bersini et al. 2014
Microfluid system	Breast cancer cells	Model created to study MDA-MB-231 breast cancer cell extravasation into an actively secreting bone mimicking microenvironment generated with osteo-differentiated hBM-MSCs through perfusable human microvascular networks composed of endothelial and mural-like cells.	The model demonstrated its effectiveness as a drug screening assay, being able to investigate the antimetastatic role of adenosine in a human bone-mimicking microenvironment.	- Presence of a microvascular network; - Analysis of cell attachment under more relevant dynamic conditions.	- Difficult to separate to different cell types; - No similarities with native bone microenvironments; - Absent 3D cell-matrix interaction; - No hypoxic setting; - Difficult to reproduce.	Jeon et al. 2015
Microfluid system	Prostate cancer cells	Tri-culture of PC-3 prostate cancer cells, osteoblasts (MC3T3-E1) and HUVECs.	Formation of uniformly-sized spheroids in co-culture.	- Biochemically and biophysically controlled 3D microenvironments.	- Difficult to separate to different cell types; - No similarities to the native bone microenvironments; - Absent 3D cell-matrix interaction; - No hypoxic setting; - Difficult to reproduce.	Hsiao et al. 2009

### Bioreactors

To facilitate accelerated cell growth and to maintain unrestricted cell-cell interactions, a rotating wall vessel (RWV) bioreactor was originally designed to mimic certain conditions occurring in the microgravity environment of space. This was then adapted by Sung et al. [40]. It allowed examine possible permanent morphological, genetic and behavioral changes in bone stromal cells after being exposed to either androgen-dependent LN cells or androgen-independent and bone metastatic C4-2 prostate cancer cells. The authors co-cultured MG63, a human osteosarcoma cell line, and HS27A, a normal immortalized human osteoblastic cell line, with LN and C4-2 prostate cancer cells and the derivate bone stromal cells. Their results highlighted that both normal and osteosarcoma bone stromal fibroblasts co-cultured with human C4-2 prostate cancer cells underwent permanent morphological changes. The resultant stromal fibroblasts showed permanently altered cytogenetic features, gene expression profiles, and growth inductive-properties. These findings underlined the ability of the transitioned stromal cells to induce, under 3D condition, prostate cancer growth and metastasis [40]. While the RWV system minimizes physical forces, other bioreactors are designed to model naturally occurring forces in the tumor microenvironment. Bioreactors have also been designed to enable the investigation of cancer cell interactions with stromal cells [41–44]. The importance of these interactions is highlighted in direct co-culture studies where cancer cells and osteoblasts were in contact [41–44]. A compartmentalized bioreactor was utilized by several authors for the growth of osteoblastic tissue (OT) and the co-culture of OT with metastatic breast cancer cells [41–43]. In this set-up, osteoblastic cells are cultured in one compartment and separated by a cellulose membrane from the medium compartment. The membrane allows for continuous exchange of nutrients and waste products, while cell-secreted macromolecules stay within the culture compartment [42]. The bioreactor-based co-culture system allows the mechanistic study of the early stages of metastatic colonization of breast cancer cells on bone tissue. As shown by Dhurjati et al. and by Krishnan et al., murine derived osteoblastic tissue, generated from a bioreactor, showed bone tissue-like characteristics by expressing bone matrix protein [41, 43]. They also reported that MDA-MB-231 breast cancer cells formed colonies and were able to penetrate the murine OT when co-cultured in a bioreactor [41, 43]. The sequential stages of the interaction between invasive cancer cells and OT, including cancer cell adhesion, penetration and colony formation, reflected some of the features involved in breast cancer bone metastasis observed in clinical practice [41–44]. In addition, Krishnan et al. [44] implemented the specialized culture system utilized by Dhurjati et al., Mastro et al. and by their own group [41–43] to evaluate whether 3D mineralizing tissue, derived from co-cultures of osteoblasts and osteoclasts in the bioreactor, could be a relevant *in vitro* bone surrogate for studying the early stages of breast cancer colonization. They created a tri-culture system, consisting of the major cell types responsible for metastatic cancer in bone and were able to provide morphological and molecular evidence that breast cancer cells degrade OT *via* osteoclast stimulation. In fact, when MDA-MB-231 breast cancer cells were introduced in osteoblastic-osteoclastic co-culture, breast cancer cells migrated toward sites of active remodeling and clustered as an aggregation of cells that further degraded the osteoblast matrix. Particularly, breast cancer cells appeared to undergo chemotaxis toward active areas of osteoclast activity. They proliferated to form colonies that were a combination of osteoclasts, cancer cells and putative pre-osteoclasts [44]. All these models [41–44] allow a detailed study of fundamental osteobiological and osteopathological processes in a way that will enhance the development of therapeutic interventions for skeletal metastasis. Recently, Krishnan et al. [45] have successfully grown mouse melanoma cells, human prostate cells and mouse myeloma cells in co-culture in a bioreactor with MC3T3-E1 osteoblasts and each of these different lines displayed a characteristic growth pattern. They have also tested the replacement of osteoblasts with endothelial and stromal cells in the bioreactor, showing a rapid growth of these cells. However, when they added MDA-MB-231 cells in co-culture with stromal and endothelial cells they did not detect the same patterns of growth as with osteoblasts co-culture [45].

### Microfluid device

Microfluidic technologies offer a number of useful capabilities for analysis, including the ability to use very small quantities of sample and reagent and to perform experiments with short processing times, high resolution and high sensitivity. Microfluidics technologies overcome some of the technical limitations of traditional assays [46], allowing the study of cancer metastases under biochemically and biophysically controlled 3D microenvironments, coupled with high-resolution real-time imaging. A microfluid 3D culture model, consisting of 3 media channels and 4 independent gel channels, has been developed to analyze the specificity of human breast cancer bone metastasis [46]. By recreating a vascularized osteo-cell conditioned microenvironment, using bone marrow derived mesenchymal stem cells (hBM-MSCs) and endothelial cells (HUVECs), the authors identified molecular pathways critical for the extravasation of breast cancer cells, involving the breast cancer cell surface receptor CXCR2 and the bone-secreted chemokine CXCL5 [46]. However, in this system, the vascular wall was represented by an endothelial monolayer. Jeon et al [47] set-up an organ specific human 3D microfluid model that enables the study of human metastatic breast cancer cell extravasation within a perfusable human microvascularized bone-mimicking microenvironment. A triculture of primary hBM-MSCs, osteodifferentiated primary hBM-MSCs and HUVECs was embedded in a fibrin gel to generate a microvascular network enclosed in a bone-mimicking microenvironment matrix. This was characterized by actively secreting osteodifferentiated cells, which created naturally formed molecular gradients affecting both microvasculature and cancer cells. The microfluidic model, containing a microvascular network used for organ specific extravasation experiments, was characterized by anastomoses with the lateral media channels. MDA-MB-231 breast cancer cells were introduced and extravasation events monitored. The study demonstrated also the effectiveness of this model as a drug screening assay, being able to investigate the antimetastatic role of adenosine in the human bone-mimicking microenvironment. The importance of cellular interaction between cancer cells and bone stromal cells is highlighted also by Hsiao et al. [48] that engineered a two-layer microfluid system to culture a 3D multi-cell type spheroid containing PC-3 metastatic prostate cancer cells, osteoblasts and endothelial cells. This microfluid device ensures the incorporation of all co-culture cell types into each spheroid and keeps the spheroids stationary for at least a week. This allows an easy tracking of individual spheroids and of PC-3 cells inside them. The engineered 3D microfluidic tumor model mimics the bone microenvironment where the metastatic prostate cancer cells are in. This platform greatly decreased the proliferation rate of PC-3 cells without reducing viability and may more faithfully mimic the *in vivo* growth behavior of malignant cancer cells within bone metastatic prostate cancer.

## MATRIX-ASSISTED ASSEMBLY OF BONE METASTASES MODELS

Advancement in tissue engineering technology platforms has enabled researchers to create matrix-derived 3D metastasis models that more closely recapitulate the pathophysiological features of native metastatic tissues. Thus, in this section we summarize several types of matrices, natural or synthetic, that have been employed for the assembly of 3D bone metastasis models (Table 2).

**Table 2:** 3D matrix-assisted assembly models of bone metastasis

3D model	Cancer cell type	Experimental design	Main results	Advantages	Disadvantages	Reference
Naturally derived matrices	Prostate cancer cells	Prostate cancer cells (PC-3 and LNCaP) cultured on collagen-based scaffolds (collagen with glycosaminoglycan or collagen with different levels of nano-hydroxyapatite, HA).	PC-3 cells cultured on 3D collagen-based scaffolds resulted in reduced levels of metalloproteinases; elevated levels of prostate specific antigen (PSA) in LNCaP cells cultured on 3D collagen-based scaffolds.	- Scaffolds with a pore structure that facilitates the infiltration of cells and nutrients; - Easily reproducible.	- Absent 3D cell-matrix interaction; - No similarities to the native bone microenvironments; - No hypoxic setting.	Fitzgerald et al. 2015
Naturally derived matrices	Breast cancer cells	Co-culture of MDA-MB-231 breast cancer cells, human osteoblasts-like cells (MG63) and MSC using non-mulberry A. mylitta fibroin scaffolds.	The interaction of cancer cells with the bone microenvironment varies with spatial organization, presence of osteogenic factors and stromal cell type; co-culture with cancer cells decreases the population of osteoblast-like cells and mineralization of extracellular matrix, increases drug resistance, invasiveness and angiogenicity.	- Scaffolds structurally more resistant to protease degradation; - Scaffold with highly porosity; - Scaffold naturally posses Arg-Gly-Asp sequences; - Presence 3D cell-matrix interaction; - Easily reproducible.	- Short experimental time; - No hypoxic setting.	Talukdar et al. 2013
Naturally derived matrices	Breast cancer cells	Mouse mammary adenocarcinoma cells (4T1) on 3D collagen-glycosaminoglycan scaffolds with or without BMP-2.	BMP-2 induces osteomimicry at the metastatic site, promotes the formation of microcalcifications in the breast and improves the mineralization of 4T1 cells in osteoblast cultures.	- Identification of the component essential for mineralization; - Scaffold with highly porosity; - Easily reproducible.	- The hydroxyapatite calcifications could potentially aggravate tumor growth; - Absent 3D cell-matrix interaction; - No hypoxic setting.	Cox et al. 2012
Naturally derived matrices	Breast cancer cells	Co-culture of breast cancer cells and BM-MSCs in a 3D collagen biomatrix.	The cell-cycle arrest of breast cancer cells is reversible either changing the microenvironment or inhibiting the signaling pathways; breast cancer cells retain their ability to proliferate.	- Useful to investigate the mechanisms that control dormancy of cancer cells; - Easily reproducible.	- Lack of complexity and capture a limited number of interactions between few cellular components at the metastatic site or between cancer cells and the ECM; - No hypoxic setting.	Marlow et al. 2013
Naturally derived matrices	Prostate cancer cells	Cultures of PC-3 and BMP-2 coupled on 3D silk fibroin scaffolds.	BMP-2 stimulates the migration of PC-3 cells; gene expression by PC-3 cells in scaffolds coupled with BMP-2 significantly increases when compared to transcript expression in the unmodified silk scaffolds; increases expression of Wnt 7B.	- Ability to control inputs and outputs to and from the system; - Specific interaction with detailed osteogenic growth factors; - Easily reproducible.	- Absent 3D cell-matrix interaction; - Resemblance of scaffold stiffness to bone is questionable; - No hypoxic setting.	Kwon et al. 2010
Synthetic matrices	Prostate cancer cells	Cancer cells C4-2B cultured on electrospun poly (ε-caprolactone) (PCL) fibers and PCL/gelatin composite scaffolds modified with PlnDIV.	The peptide increases the proliferation of C4-2B cells, reduces the expression of tight junction protein and increases the focal adhesion kinase phosphorylation on tyrosine 397.	- Incorporation of the peptide into electrospun matrix is a key improvement to create a successful 3-D pharmacokinetic cancer model.	- Absent 3D cell-matrix interaction; - Electrospun PCL fibers require additional surface modification due to lack of functional groups; - No hypoxic setting; - Difficult to reproduce.	Hartman et al. 2010
Synthetic matrices	Prostate cancer cells	Co-culture of LNCaP prostate cancer cells embedded within PEG hydrogels, or LNCaP and PC-3 with hOBs, within a TEB based on mPCL-TCP.	The intercellular and prostate cancer cell-bone matrix interactions lead to elevated levels of matrix metalloproteinases, steroidogenic enzymes and PSA.	- Similarities with the bone-like microenvironment; - Model practicable and versatile for studying intercellular and cell-matrix interaction at a cellular and molecular level.	- Prostate cancer cells gene expression cannot be analyzed separately from hOBs; - No hypoxic setting; - Difficult to reproduce.	Sieh et al. 2010; Sieh et al. 2014
Synthetic matrices	Breast cancer cells	MDA-MB231 breast cancer cells cultured within non-mineralized and mineralized inorganic polymeric scaffolds composed of PLG and HA particles.	Tumor cell adhesion, proliferation, and secretion of pro-osteoclastic interleukin-8 (IL-8) increase in mineralized scaffolds compared to non-mineralized scaffolds; supernatants of MDA-MB-231 cell cultures collected on mineralized scaffolds promote osteoclastogenesis in an IL-8 dependent manner.	- Stiffness of scaffold comparable to bone environment; - Investigation of the prometastatic role of HA; - Easily reproducible.	- Absence of bone stromal cells; - No hypoxic setting.	Pathi et al. 2010
Synthetic matrices	Breast cancer cells	Co-culture of hBM-MSCs and breast cancer cells on 3D porous chitosan bone scaffolds containing HA.	Breast cancer cells adhesion and proliferation increase with decreasing HA particle size and concentration; MSCs upregulate the expression of the well-known metastasis-associated gene metadherin within breast cancer cells.	- Presence of bone stromal cells; - Specific interaction with detailed osteogenic growth factors; - Use of several cancer cells with different metastatic activity; - Easily reproducible.	- Insufficient regarding a direct cell-cell communication; - No hypoxic setting.	Zhu et al. 2014
Synthetic matrices	Renal carcinoma cells	Bone-derived human 786-O RCC cultured in a 3D hyaluronate-based hydrogel system.	RCC spheroids in 3D hydrogels demonstrate lower proliferation rates compared to their counterparts grown in 2D; Cad11 and CXCR4 more closely mimic the growth rate observed in vivo; bone-derived human 786-O RCC cells proliferate and survive long term in these hydrogels.	- Hydrogels provide a microenvironment more similar to the in vivo bone metastatic microenvironment; - Easily reproducible.	- Insufficient regarding a direct cell-cell communication; - No hypoxic setting.	Pan et al. 2015

### Naturally derived matrices

In order to investigate the conditions inducing an osteomimetic response of breast or prostate cancer cells (4T1, PC-3 and LNCaP), different 3D collagen- scaffolds (collagen-glycosaminoglycan or collagen with different levels of nano-hydroxyapatite, HA) have been used [49–50] demonstrating that both scaffolds successfully supported breast or prostate cancer cell infiltration, growth and viability. In addition, Cox et al. demonstrated that the presence of bone morphogenic protein 2 (BMP-2) further enhanced *in vitro* mineralization of breast cancer cells [49]. In order to facilitate the study of the dynamic interaction between breast cancer cells and several cell types present in bone marrow stroma, Marlow et al. [51] established a novel experimental systems able to model the bone microenvironment of the breast cancer metastatic niche. This system was based on a 3D-collagen biomatrix seeded either with human primary BM-MSCs or immortalized lines representing cell types found in human bone marrow: osteoblasts (human fetal osteoblasts, hFOBs), mesenchymal cells of bone marrow origin (HS-5), and endothelial cells, in a mix. In order to substitute conventional natural polymers (e.g. collagen) silk-based biomaterials have also been used by some authors [52–53]. Using non-mulberry A. mylitta fibroin scaffolds Talukdar et al. [53] developed a co-culture based metastasis model to study interactions between MDA-MB-231 breast cancer cell line, human osteoblast-like cell line and mesenchymal stem cells. They found that breast cancer cells were able to proliferate and migrate through the porous material and to form clusters. Despite the absence of bone stromal cells, specific roles of bone components with regards to cancer progression were also defined by Known et al. [52], demonstrating that silk fibroin scaffolds coupled with BMP-2 stimulated the migration of PC3 cells. Moreover, gene expression by PC3 cells in scaffolds coupled with BMP-2 was also significantly increased when compared to transcript expression in unmodified silk scaffolds, suggesting cell stimulation by BMP-2. Osteogenic marker expression and Wnt 7B expression support the hypothesis that prostate cancer cells have bone cell-like properties to survive, proliferate, migrate and invade the bone environment.

#### Synthetic matrices

To gain mechanistic understandings of cancer metastasis, several authors used 3D polymeric scaffolds as innovative tools for recreating microenvironmental conditions in culture [54–57]. Sieh et al. [54] cultured PC3 and LNCaP prostate cancer cells on a polycaprolcatone-tricalcium phosphate (mPCL-TCP) bone-mimetic composite scaffold, fabricated by wrapping a human osteoblast cell sheet around a cell-seed. The authors found that intercellular and prostate cancer cell-bone matrix interactions contributed to the observed increase in the expression of various biomarkers associated with prostate cancer cells bone metastasis. Additionally, the authors [55], using the same construct, established an indirect 3D *in vitro* co-culture model to study paracrine interactions between prostate cancer cells and human osteoblasts (hOBs). This model consisted of LNCaP prostate cancer cells embedded within polyethylene glycol (PEG) hydrogel and with hOBs cultured with the construct. In the PEG hydrogel, LNCaP cells form a multicellular mass that resembles a vascular tumor, while the construct provides the structure in which hOBs secrete factors that can be influenced by cancer cells. It was demonstrated that the paracrine interaction between LNCaP cells and hOBs influences LNCaP cell growth, gene expression and protein synthesis, as demonstrated in a previous study [54]. In addition, Hartman et al. demonstrated that the modification of an electrospun poly (ε-caprolactone) (PCL) fiber and PCL/gelatin composite scaffold with perlecan domain IV (PlnDIV) peptide, supported key signaling events leading to proliferation, survival, and migration of C4-2B prostate cancer cells [56]. HA is the main extracellular matrix component of human bone. For this reason in order to investigate the pro-metastatic role of HA by culturing MDA-MB-231 breast cancer cells, a 3D inorganic polymeric scaffold (polylactide-coglycolide, PLG) containing HA particles, fabricated using a gas foaming-particulate leaching technique, was engineered [57]. The authors demonstrated that HA controls several key steps of breast cancer bone metastases and that interleukin-8 (IL-8) could play an important role in this process by directing mammary tumor cells towards a phenotype that promotes secondary tumor growth and bone destruction. Recently, Zhu et al. [58] incorporated HA, of varying size and crystallinity, in a 3D porous chitosan bone scaffold. hBM-MSCs were used to deposit bioactive factors within the bone scaffold, and further grow MSCs within the scaffold in order to create a more biomimetic microenvironment. Three human breast cancer cell lines, with different metastatic activity (MDA-MB-231, MCF-7 and transfected MDA-MB-231) were cultured with this 3D bone model showing that breast cancer cell adhesion and proliferation increased when reducing HA particle size and concentration. In addition, the co-culture of MSCs and MDA-MB-231 in this bone model revealed that MSCs have the capacity to upregulate the expression of the well-known metastasis-associated gene metadherin within breast cancer cells. Differently from the above mentioned studies, Pan and coworkers fabricated a 3D hyaluronate-based hydrogel in order to study renal carcinoma cells (RCC) bone metastasis [59]. The bone-derived human 786-O RCC subline proliferated and survived long term in the 3D hyaluronate hydrogel-based scaffold. Overall, gene expression patterns of RCC spheroids in 3D, more closely mimicked those observed *in vivo* providing an improved platform for RCC bone metastasis studies.

## DIRECT BONE-TUMOR CELL CONTACT

In contrast with more simplistic models, an attractive physiological approach able to mimic bone metastasis may be the culture of bone tissue explants that give the opportunity to study tumor cells in a natural microenvironment, thus including all bone cell types as well as the extracellular matrix (Table 3).

**Table 3:** 3D direct bone tumor cell contact models of bone metastasis

3D model	Cancer cell type	Experimental design	Main results	Advantages	Disadvantages	Reference
Direct bone-tumor cell contact	Prostate cancer cell line	Dissected calvarial bones from inbred mice cultured on metal grids with prostate cancer cells (PC-3, osteolytic phenotype, or LNCaP, mixed/osteoblastic phenotype) in a two-compartment in vitro co-culture model.	Co-culture of calvarial with human PC-3 cells resulted in increased transcription of gene associated with the activation and function of osteoclasts, while when LNCaP were used there was a shift to a predominantly osteoblastic gene expression pattern.	- Natural heterogeneity of bone cell population within bone; - Easily reproducible.	- No hypoxic setting; - Leaves out the aspect of species-specific osteotrophism.	Nordstrand et al. 2009
Direct bone-tumor cell contact	Prostate and breast cancer cells	Free-floating live mouse calvarial bone co-culture with different cancer cell lines (breast tumor cells lines, MCF-7 and MDA-MB-231, and prostate tumor cell lines, LNCaP Clone FGC and PC-3), in a roller tube system under hypoxic conditions.	Cancer cells showed a remarkable affinity and specificity for “endosteal side” of the bone where they colonized and proliferates. This was concurrent with the differentiation of resident stem/progenitor cells to osteoclasts and bone resorption. In contrast, under bone formation conditions this model revealed different pathophysiology where breast cancer cells continued to induce osteoclastic bone resorption whereas prostate cancer cells led to osteoblastic bone formation.	- Hypoxic conditions; - Specifically defined bone remodeling stage; - Presence of multicellular component; - Easily reproducible.	- Leaves out the aspect of species-specific osteotrophism; - Short experimental time.	Curtin et al. 2012
Direct bone-tumor cell contact	Breast cancer cells	Co-culture of viable human subchondral bone discs with MDA-MB-231 or T47D human breast tumor cells.	The in vitro inoculation of breast cancer cells colonized human bone cores remaining viable for up 4 weeks.	- Species-specific osteotrophism; - Easily reproducible.	- No hypoxic setting; - The primary aim was to set-up of an in vivo study.	Holen et al. 2015

List of abbreviations used in the tables: Rotating wall vessel (RWV), osteoblastic tissue (OT), bone marrow-derived human mesenchymal stem cells (hBM-MSCs), human umbilical vein endothelial cell (HUVECs), prostate specific antigen (PSA), hydroxyapatite (HA), mesenchymal stem cells (MSC), bone mineral protein (BMP), extracellular matrix (ECM), electrospun poly (ε caprolactone) (PCL), perlecan domain IV peptide (PlnDIV), polyethylene glycol hydrogels (PEG), human osteoblasts (hOBs), tissue engineered bone construct (TEB), polycaprolactone-tricalcium phosphate (mPCL-TCP), polylactide-coglycolide (PLG), interleukin-8 (IL-8), renal carcinoma cells (RCC).

Nordstrand et al. [60] implemented a murine calvarial explant to monitor how tumor cells influenced the bone remodeling process and how bone microenvironment influenced tumor cells. To achieve this, they established a two-compartment *in vitro* co-culture model using different prostate cancer cell lines (PC3, osteolytic phenotype, and LNCaP, mixed/osteoblastic phenotype) and followed the trans-activation of bone and/or tumor cells. In order to target prostate cancer bone metastases, dissected calvarial bones from inbred mice were cultured on metal grids with prostate cancer cells forming a two-compartment model, which allows for paracrine signaling between the two-compartments. Co-culture mouse calvariae with human PC-3 cells resulted in increased transcription of genes associated with the activation and function of osteoclasts, while when LNCaP were used there was a shift to a predominantly osteoblastic gene expression pattern. Since it is widely known that tumor biology research have benefited from the hypoxia model which mimics nutrient and oxygen insufficiency at the tumor-host interaction, Curtin et al. [61] developed an *ex-vivo* 3D cancer bone metastasis model composed of free-floating live mouse calvarial bone, in presence of different human cancer cell lines (breast tumor cells lines, MCF-7 and MDA-MB-231, and prostate tumor cell lines, LnCap Clone FGC and PC3), in a roller tube system under hypoxic conditions. The use of this model revealed remarkable specificity of cancer cell colonization and growth, predominantly on the endosteum layer of bone, which contains stem/progenitor cells for osteoclasts differentiation. Bone resorption is exerted strictly by differentiated osteoclasts and even when the mineralized bone was completely dissolved, cancer cells remained on the surface of the endosteal layer with no migration beyond this cell layer. This aspect reflects the specific affinity and signals from the bone endosteal cell population, which is critical for cancer cells homing to bone and for the attachment and colonization of bone microenvironments. However, despite this 3D *in vitro* model can mimic the *in vivo* condition, it leaves out the considerable aspect of species-specific osteotrophism. In fact, Holen et al. [62] highlighted that human breast cancer cells preferentially home to human bone fragments implanted in mice, thus underlining their species-specific behavior. Before the development of the *in vivo* study, they set-up a 3D *in vitro* model by culturing viable human subchondral bone discs with human breast tumor cells (MDA-MB-231 or T47D) and revealing that the *in vitro* inoculation of breast cancer cells colonized human bone cores and remained viable for up to 4 weeks.

## DISCUSSION AND FUTURE DIRECTION

Realizing the limits of monolayer cultures, and stimulated by the complexity of the native tumor microenvironment, researchers developed several simple and complex (with added stromal component) 3D *in vitro* models that recapitulate certain features of tumor tissues. In fact, given the growing interest in this field (PubMed citations for this topic have increased three-fold in the 2015-2010 period compared to 2005-2010), many stimulating ideas about the use and improvement of these 3D *in vitro* models have emerged in the last decade. The main advantage of these models is the possibility to grow one or more cell types or a tissue in 3D, which reproduce *in vivo* conditions more closely. This allows to test many samples and a better control of external culture factors, which can give more reproducible results compared to those obtained *in vivo*. In addition, these models follow the 3R principles aimed at replacing/reducing/refining animal use. Thus, 3D models could provide an attractive alternative to animal models for ethical but also for economic reasons. However, despite the towering interest in the development of 3D *in vitro* models, few models have been devised to recapitulate bone metastases as yet. This review is intended to summarize and highlight the research focused on the set-up of the 3D *in vitro* model for bone metastasis looking at three fields: 1) custom designed culture devices, 2) naturally- or synthetic- derived matrices and 3) direct bone-tumor cell contact cultures. As reported above, each of the presented models has certain advantages and disadvantages. In summary, most studies (11 papers out of 23) involve the use of biologically-derived or biomimetic matrices. In detail, 5 studies were on naturally-derived matrices, while 6 were on synthetic matrices. Despite being an attractive alternative, devices able to maximize cell-cell interactions and solute transport without the adverse interference of scaffolding materials, were used in a fewer number of papers (9 papers out of 23). Among them, 6 studies used a bioreactor in order to miniaturize the natural counterparts, to introduce relevant forces or to create a controlled environment to foster the assembly of tumor-like tissues. The remaining 3 studies used the microfluidic technology that offer a number of useful capabilities for analysis, including the ability to use very small quantities of sample and reagent and to perform experiments with short processing times, high resolution and high sensitivity. Despite one of the attractive physiological approaches would seem to be the direct bone-tumor cell culture, because it allows 3D architecture together with the preservation of tissue extracellular matrix and cellular complexity, only 3 papers out of 23 reported its use. Probably this limited use is due to the fact that these models are based on the supply of fresh bone material. As shown in some of the examined studies, the use of murine bone is preferred, although its properties may be dissimilar from human bone in terms of composition and of intercellular interactions. In fact, Kuperwasser et al, using an *in vivo* model, indicated that human breast cancer cells preferentially home to human bone fragments implanted in mice, thus underlining a species-specific osteotrophism [63]. In addition, the use of human bone and human cells would allow the study of patient factors that influence the development of bone metastases, i.e. age, sex, concomitant comorbidity and unhealthy life style. Nevertheless, this review revealed that there is a lack of 3D models with the use of human bone.

Despite substantial and continued success in creating 3D bone metastasis models, several challenges and limitations still remain. For the design of more complex and physiologically more relevant microenvironments, different directions should be considered. 1) The majority of the *in vitro* models discussed here have been set-up to model bone colonization. On the contrary, there are only few models that are focused on other steps of the bone metastatic cascade, such as the extravasation of cancer cells in the bone microenvironment. The recreation of the complex, metastasis-associated vascular system *in vitro* is an essential step since these abnormal blood vessels not only influence metastasis progression, but also greatly affect drug transport within metastatic tissues [64]. 2) Many of the examined extracellular matrix embedded models are still simplistic and they do not include all the cell types and the extracellular matrix components which would make the models organotypic. This is an essential point because this extracellular-matrix is not just an inert material providing a 3D scaffold for tumor cells to grow and invade, but it also plays an essential role in the differentiation and maintenance of the tissue itself. Moreover, the extracellular-matrix has been shown to provide survival and drug resistance signals in cancer [65]. 3). Despite the bone microenvironment is hypoxic for definition with oxygen levels below 10%, only one of the examined studies considered this aspect using low-oxygen culture conditions [61]. Hypoxic conditions stimulate blood cell proliferation and blood vessel formation, and modulate the expression of extracellular matrix components and remodeling enzymes, thereby maintaining tissue homeostasis. In addition, hypoxia is present in all solid tumors over 1 cm^3^, and it is clinically associated with metastasis and poor patient outcome. As shown in the study of Curtin et al. this culture condition can be relatively easily obtained for any of the described models, using modified cell culture incubators or even simpler methods that allow a more physiological reduction of the normal oxygen concentrations to levels between 1 and 5%. 4) Although the use of murine cell lines has advantages, such as the separate analysis of the different cell populations, and the possible combination with gene knockout strategies for functional studies, the use of murine tissue may introduce species-related biases, hampering the translation of the data to human disease. Therefore the question about which source of cells (human *vs*. animal) is needed to produce data that can be predictive of clinical settings is still open. 5) For tissue engineered constructs an important biological feature would be maintaining cell viability and function over time, but most of the examined studies had a short experimental time. For smaller constructs, this may be affected by the co-culture with other cells, while for larger, printed organ technologies these concerns may center on diffusion limitations and construct perfusion. 6) Despite synthetic scaffold have a great potential to mimic several aspects of bone metastatic microenvironment, they will never be natural matrices and therefore may be more useful for investigating of individual and specific tumorigenic steps.

Despite the limitations associated with 3D bone metastasis models represent a barrier to fully mimic the native metastatic microenvironment, and consequently test effective anti-metastatic therapies, considerable progresses have been made in the attempt to create 3D *in vitro* models that are more representative of metastasis complexity. The 3D *in vitro* models reviewed here offer a realistic and controllable microenvironment that better clarifies the mechanisms that support bone invasion and metastasis. This is a key factor for 3D models since the metastatic process can be successful only if the 3D microenvironment is favorable for tumor cell invasion, metastatic dissemination and metastatic growth. Although these 3D models are becoming progressively more similar to the *in vivo* situation it is important to note that the translation of any finding into human disease models may not be easy. Therefore it is essential to have a good understanding of the intricate intra- and intercellular signaling circuits underlying the communication between the various cell types populating a metastatic tissue, and of the systemic and local factors that form the metastatic microenvironment. Direct bone-tumor cell contact models maintain the production, degradation and replacement of the matrix by osteoblasts. This, together with the presence of bone marrow with its hematopoietic stem cells (which provide white and red blood cells to the vasculature) and adipocytes, is important to investigate more in detail just the intra- and intercellular interaction and the systemic and local factors naturally occurring during bone metastases. Additionally, the use of human bone within this model, will also allow to perform more realistic *in vitro* drug testing assays, where different compounds can be evaluated in parallel. To date the success rate of anti-cancer therapy translating from *in vitro* culture systems into the clinical practice is about 5% [66]. This highlights the current relevant limitation that is probably due to the fact that efficient drug testing will need *in vitro* 3D bone metastasis models where capillary beds, stromal cells, immune system components and mechanotransduction signaling are present.

In conclusion an important question still remains: “Are these progress on 3D *in vitro* models sufficient to be replaced with advanced models?” In our opinion: “not yet”. To our knowledge, these advanced and alternative models should be characterized, tested and refined in more detail by multidisciplinary experts and, finally, they should be validated with an appropriate retrospective analysis for their ability to be predictive of the clinical outcome. In fact, these 3D models do not still entirely recapitulate the metastatic microenvironment found in clinical trials; most of them are only morphologically similar to native metastatic tissues. Phenotypic similarity and heterogeneity need to be further delineated. Thus, since no 3D *in vitro* models can completely reproduce the full complexity of bone metastases in humans, and each model has native strength and weakness, researchers could try to exploit a combination of different models. It can be expected that future studies will attempt to reproduce the complexity of *in vitro* generated bone metastatic microenvironments. These will hopefully offer a rational basis for the set-up of clinical studies that may open new strategies to deal with bone metastatic disease.
